# The association of socioeconomic status with the success of chat-based online counseling for children and youth: A latent change score modeling approach

**DOI:** 10.1016/j.invent.2024.100753

**Published:** 2024-06-14

**Authors:** Franziska Rarey, Julia Thomas, Anne Berghöfer, Lars Kuchinke, Gunther Meinlschmidt, Christine Rummel-Kluge, Richard Wundrack, Matthias Ziegler

**Affiliations:** a Humboldt-Universität zu Berlin, Department of Psychology, 12489 Berlin, Germany; b Division of Clinical Psychology and Epidemiology, Department of Psychology, University of Basel, Basel, Switzerland; c krisenchat gGmbH, Germany; d Charité Berlin, Germany; e International Psychoanalytic University Berlin, Germany; f Division of Clinical Psychology and Cognitive Behavioural Therapy, International Psychoanalytic University (IPU) Berlin, Berlin, Germany; g Department of Psychiatry and Psychotherapy, Leipzig University Medical Center, Leipzig, Germany; h Humboldt-Universität zu Berlin, Germany; i Department of Digital and Blended Psychosomatics and Psychotherapy, Psychosomatic Medicine, University Hospital and University of Basel, Basel, Switzerland; j Clinical Psychology and Psychotherapy - Methods and Approaches, Department of Psychology, Trier University, Trier, Germany

**Keywords:** Adolescence, Latent change score modeling, Online counseling, SES

## Abstract

Children and youth from lower subjective socioeconomic status (SES) backgrounds are at a heightened risk of mental disorders. Online counseling is a valuable tool to reach those less likely to seek professional help, but its success across different SES backgrounds remains unclear. This study explores the association between subjective SES and online counseling outcomes. Children and youth (*N* = 2139) between 10 and 24 years-of-age received chat-based online counseling and reported on SES, negative feelings before and after the chat, and perceived helpfulness of the chat via an online assessment tool. The results of a latent change score model showed a significant association between SES and negative feelings before chatting, indicating that lower SES predicted more negative feelings (*r* = −0.26, *p* < .001). Further, SES was indirectly associated with the change in negative feelings from before to after counseling, mediated by the extent of negative feelings before the chat (*β* = 0.07, 95%CFI = [0.05–0.10]). Current findings extend research on online counseling programs in the context of SES. Despite higher counseling needs among low SES individuals, they do not benefit proportionally from existing online services in this sample. Future research should investigate barriers to help-seeking and implement specialized counselor training programs.

## Highlights

• Children and youth with low subjective socioeconomic status (SES) face an increased risk of mental disorders.
• Online counseling is a valuable tool to reach those less likely to seek help, but its success across different SES backgrounds remains unclear.
• Children and youth (*N* = 2,139) 10-24 years-of-age received chat-based online counseling and reported on SES, negative feelings, and helpfulness.
• Despite higher counseling needs among low SES individuals, they do not benefit proportionally from existing online services in this sample.
• Future research should investigate barriers to help-seeking and implement specialized counselor training programs.

## 1 Introduction

As of 2021, one in five German children under 18 faced a risk of living in poverty (Funcke & Menne, 2023). These children from families with low socioeconomic status (SES) experience disadvantages in almost all areas of life, such as education, physical and mental health, and social participation (Funcke and Menne, 2023). SES is defined as an indicator combining income, education and social prestige. It marks the position of a group or an individual on the socioeconomic scale. For clarity, SES will henceforth be used to refer to ‘subjective socioeconomic status’, a measure to assess relative deprivation.

Mental health and SES are negatively associated, which has consistently been observed in most developed countries. Repeatedly, studies demonstrated that those at the bottom end of the social hierarchy suffer a disproportionately high share of the global burden of disease (Everson et al., 2002; Poulton et al., 2002; Reiss, 2013; Williams et al., 2010; Willson et al., 2007). E.g., children in families with low SES were 2.5 times more likely to have mental health issues (Klasen et al., 2017). Scholars propose that children's dependency on the environment renders them systemically more vulnerable (McCloskey et al., 1995).

The German “Healthcare Supply Report” (2018) reported an overall increase of 23 % to 28 % in mental disorder prevalences among children and youth (Steffen et al., 2018). However, <50 % of affected individuals receive adequate help (Bundes-Psychotherapeuten-Kammer, n.d.). Especially so in low-SES families, who already show the highest risk of developing mental health problems, are least likely to reach out to healthcare professionals (Klasen et al., 2017; Bundes-Psychotherapeuten-Kammer, n.d.). For instance, supposedly because of a lack of education and social measures, they have less access to health-promoting resources such as quality food and medical care and less knowledge about when and how to seek professional help (Bundes-Psychotherapeuten-Kammer, n.d.; Link and Phelan, 1995). A meta-analysis from 2018 concluded that socioeconomic deprivation is associated with poorer treatment outcomes, such as a lower chance of response to the treatment and lower remission rates (Finegan et al., 2018). In addition, low SES has been determined as a risk factor for premature termination of psychotherapy.

New approaches aim to close this gap, with online counseling and therapy emerging as a possibility to offer low-threshold support for psychosocial crises. Furthermore, the COVID-19 pandemic has pushed preventive efforts into the digital space and has led to a significant increase in the demand for online mental health services, particularly among youth (Zangani et al., 2022).

Online counseling is defined as the intentional exchange of information intended to attenuate or solve a problem presented by a client to a counselor or mental health professional (Baker and Ray, 2011). It can be delivered as instant messaging, synchronous chat, text messaging, asynchronous e-mail, and video conferencing (Barak et al., 2009; Barnett, 2005; Dowling and Rickwood, 2013). Research has shown that online counseling services are helpful and impact children's and adolescents' lives regarding somatic and mental health issues, such as subthreshold depression (Cuijpers et al., 2017) and suicidal thoughts (Kohls et al., 2022). Online counseling interventions could be an opportunity to reach children and youth who otherwise would not get any professional help (Eckert et al., 2022; Faber et al., 2023). However, research on the effectiveness of online counseling dependent on the clients' SES is still scarce.

**Fig. 1:**
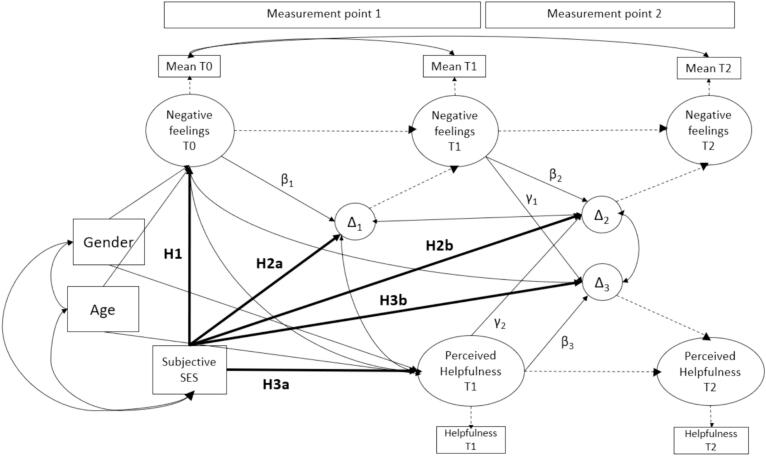
Model with all hypotheses.

The study seeks to answer the question: ‘How does SES relate to the success of chat-based online counseling for children and youth?’ It aims to formulate and test a model of online counseling success that considers SES and the severity of negative feelings experienced by the chatters. Building on the findings above, we will examine the following three hypotheses, based on data from a German crisis text line (krisenchat): First, children and youth from low SES backgrounds experience more negative feelings than their counterparts with high SES. Secondly, children and youth from low SES backgrounds decrease less in their experienced negative feelings. This will be the case a) directly after the chat and b) four weeks after the chat. This leads to the third hypothesis that a) children and youth from families with low SES perceive krisenchat as less directly after the chat, and b) they will change their rating to even less helpful after four weeks. The combination of all hypotheses and the model can be seen in Fig. 1.

## 2 Materials and methods

### 2.1 krisenchat

One online counseling program providing a service for German-speaking children and youth below the age of 25 is krisenchat. This service provides a real-time chat platform for professional, online counseling, accessible without the need for registration or payment. The service can be contacted 24/7, pseudonymously, meaning that users' telephone numbers are stored with a masked chat ID. The counselors at krisenchat are largely volunteers who are professional or in-training psychologists, psychotherapists, or social workers. They undergo a structured training program in messenger-based counseling. Its primary goal is to address children who have never reached out to seek psychological help (https://krisenchat.de/, n.d.). Counselors emphasize collaborative problem-solving, self-efficacy and co-regulation of clients in crisis, as well as referral to the secondary health care system. A high feasibility and acceptability of the service (Eckert et al., 2022) with a high recommendation rate of 89.6 % among users experiencing suicidal ideation (Kohls et al., 2022) was shown.

### 2.2 Participants

Overall, 2739 (500 male, 2079 female, 114 diverse, 46 not disclosed) German-speaking children and youth took part in the first measurement point. The mean age was *M* = 16.77 years (*SD* = 3.37, range 10–24 years). Most of the sample perceived their socioeconomic status as somewhat better than most other people in Germany (*n* = 1052, *M* = 2.88, *SD* = 0.86, range = 1–4).

### 2.3 Procedure

We collected the data from all children and youth who initiated contact with krisenchat between July 21, 2022, and April 20, 2023, by extending an invitation to participate in the study through the platform. The chatters automatically received the invitation to the first feedback questionnaire 3 h after the counseling session ended. If a participant gave permission to be recontacted, an invitation to the follow-up questionnaire was sent out via the chat platform four weeks after the first counseling session, regardless of recontact. Exclusion criteria were non-disclosure of age, gender or SES, or age above 24 or below 10 years. Thus, the final sample included in the analysis consisted of 2139 participants. All answers in the questionnaire were given voluntarily.

### 2.4 Material

The data in the present study were collected within a project funded by the Federal Ministry of Health in Germany. The analysis in this study was a secondary data analysis which received ethical approval by the ethics committee of the University of Leipzig (ZMI1-2521FEP001). The data were collected via two German questionnaires administered by krisenchat and are self-report measures.

***The questionnaires***. The first questionnaire contained the five-item *Screening Tool for Psychological Distress* (STOP-D), developed and validated by Young et al. (Young et al., 2007). The questionnaires exist in German and English and were translated by two professional translators independently. All variables used in this study are described in detail in the following.

***Negative feelings***. Negative feelings were assessed using five items from the STOP-D (Young et al., 2007). Participants were asked to rate to which extent they felt impacted by five negative feelings. The five feelings were sadness or dejection, anxiety/edginess, stress, rage, and the feeling of not getting enough support. Participants rated each feeling on a scale from 0 = not at all to 9 = severely. There are no reliability estimates for the STOP-D items. However, in terms of validity, the items show robust correlations with other measures of depressive, anxiety, or anger symptoms, such as the Beck's Depression Inventory and the State-Trait Anger Expression Inventory (Young et al., 2007).

***Perceived helpfulness***. Perceived helpfulness was assessed via a single item in the questionnaire. Participants could answer the question of whether the counseling session has helped them with their issues or not. The scale ranged from 0 = not helpful at all, to 3 = very helpful.

***SES***. In this study, SES was assessed via a single item, on a scale from 1 to 4 (1 = worse, 2 = somewhat worse, 3 = somewhat better, 4 = better), with the following, original wording: *Denke an die Bildung*, *Arbeit und das Geld*, *das deine Familie und du haben*. *Wie geht es euch im Vergleich zu den meisten anderen Menschen in Deutschland*? (English: *Think about the education*, *job*, *and money that you and your family have*. *How is your standard of living as compared to most other people in Germany*?). The single-item assessment is based on the McArthur Scale of Subjective Social Status that showed good retest reliability (Giatti et al., 2012) and robust predictive validity (Galvan et al., 2022).

***Gender and Age***. Gender and age were assessed via the chat clinical record. Options were female, male, diverse or non-disclosure.

### 2.5 Data preprocessing

**Fig. 2:**
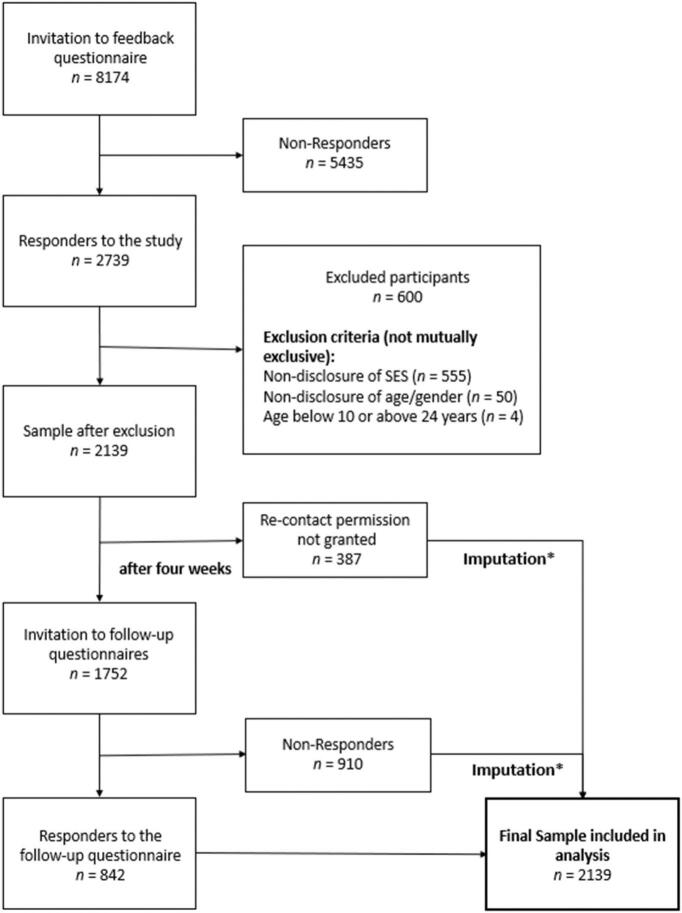
Study Flow Chart. *Missing data due to drop-out were handled in the final analysis via full information maximum likelihood. Abbrev.: SES, Socioeconomic Status.

Data were collected using the open-source online-survey tool *formr* (Arslan et al., 2020). The data were stored in an SQL database in the krisenchat data warehouse. Data retrieval was performed as an SQL query. Incompletely answered questionnaires, non-responders for gender (*n* = 42), age (*n* = 4) or SES (*n* = 555) were excluded from analysis. Overall, 2139 participants remained for the final analyses. Of those, 387 did not permit re-contact. Of the 1752 invited participants 910 did not complete the four weeks follow-up questionnaire, resulting in a non-response rate of 51.95 %. The complete study flow chart is depicted in Fig. 2.

### 2.6 Data analysis

A latent change score (LCS) model based on the work by McArdle and Hamagami (McArdle and Hamagami, 2001) with latent change variables from T0 (baseline) to T1 (directly after the chat) and T1 to T2 (four weeks follow-up) for negative feelings and from T1 to T2 for perceived helpfulness was set up.

Several advantages justify SEM's increasing popularity and superiority over single indicator-based analyses. First, latent variable modeling allows for better construct validity than a single measure (Goodwin and Goodwin, 1991) and offers more accurate estimates since it is free of error by definition (Wolf and Brown, 2013).

Most importantly, structural equation modeling allows for a highly reliable analysis of change over time. LCS models define the change in a construct over time as latent variables while simultaneously examining autoregressive trajectories (McArdle, 2009; McArdle and Hamagami, 2001). Additionally, they provide empirical evidence for the theory of dual change, e.g., two constructs sharing a dynamic relationship over time. Therefore, an LCS model perfectly fits with the purpose of this study.

T0 and T1 were assessed simultaneously at the first measurement point, meaning that T0 is a retrospective measure. T2 was assessed at the second measurement point four weeks later. All five indicators for the negative feeling construct were parceled into one indicator due to psychometrical and modeling-related benefits (Matsunaga, 2008). To examine the association of SES with the dynamic change, SES was regressed on negative feelings at T0 (H1), counseling success at T1 (H3a), and all latent change variables (H2a, H2b and H3b). Covariates, that is, age and gender, were added to the model to ensure the robustness of the effects of interest and were allowed to correlate with each other (Sladen, 1982; Thomsen et al., 2005; McRae et al., 2008; Zlomke and Hahn, 2010). The model was set up in R (*R Foundation for Statistical Computing*, 2022) using the lavaan package (Rosseel, 2012). Other packages used during the analysis were dplyr (Wickham et al., 2018), psych (Revelle, 2022), car (Fox and Weisberg, 2019), apaTables (Stanley, 2021), naniar (Tierney et al., 2023), tidyr (Wickham et al., 2023), ggplot (Wickham, 2016), and afex (Singman et al., 2016). A robust maximum likelihood estimator was used since the data violated the assumption of normal distribution (Suh, 2015). Missing data were handled in the analysis model using the full information maximum likelihood estimator. The measurement error of each parcel was fixed to their reliability estimate.

## 3 Results

### 3.1 Descriptive statistics

Table 1 displays the mean levels of negative feelings across time points, segmented by SES group. Conversely, Table 2 outlines the statistics for perceived helpfulness, categorized in the same manner. The mean scores, separated by SES group, showed that the extent of negative feelings decreased at T1 and then increased at T2, but remained below the means before chatting at T0. In addition, the group with the lowest SES showed higher means than all other three groups. A second look at Table 1 reveals the imbalance of individuals per SES group (lowest SES: *n* = 163 at T0 and T1, third SES: *n* = 1028).

**Table 1:** Mean negative feelings by SES and measurement point.

SES group	N	Mean	SD
**T0**			
Worse	163	7.00	1.41
Somewhat worse	441	6.57	1.47
Somewhat better	1028	6.34	1.54
Better	507	6.50	1.68
**T1**			
Worse	163	4.73	2.27
Somewhat worse	441	4.42	2.16
Somewhat better	1028	4.36	2.20
Better	507	4.07	2.40
**T2**			
Worse	52	6.13	1.74
Somewhat worse	161	5.52	1.75
Somewhat better	436	5.53	1.97
Better	193	5.31	2.10

Abbrev: SD, standard deviation; SES, Socioeconomic Status.

**Table 2:** Mean perceived helpfulness by SES and measurement point.

SES group	N	Mean	SD
**T1**			
Worse	163	2.21	0.77
Somewhat worse	441	2.29	0.76
Somewhat better	1028	2.36	0.68
Better	507	2.39	0.74
**T2**			
Worse	52	2.20	0.76
A bit worse	161	2.16	0.70
A bit better	436	2.27	0.73
Better	193	2.30	0.72

Abbrev: SD, standard deviation; SES, Socioeconomic Status.

ANOVAs and Chi-Square-Tests were conducted and revealed no significant differences between the composition of completers and drop-outs regarding SES and the baseline mean of negative feelings. However, completers differed significantly from drop-outs regarding age, with drop-outs being on average one year older than completers, and gender, which is most likely due to a high gender imbalance in the sample. For ANOVA and Chi-Square-Test results see Appendix A.

The mean of negative feelings correlated moderately between all three time points (0.33 < *r*s < 0.46, *p* < .01–0.05). In addition, the means of negative feelings at T1 and T2 correlated significantly negatively with the perceived helpfulness items (−0.33 < *r*s < −0.18, *p* < .01–0.05). This indicates a negative relationship between the extent of negative feelings and the rating of krisenchat as helpful, meaning a high extent of negative feelings also came with lower helpfulness ratings. Perceived helpfulness correlated strongly between both assessed time points (*r* = 0.60, *p* < .01). There were also three negative correlations of SES with the mean of negative feelings at baseline (*r* = −0.13, *p* < .01) and directly after the chat (*r* = − 0.07, *p* < .01) and four weeks later (*r* = −0.10, *p* < .01). SES correlated weakly positive with the perceived helpfulness directly after the chat (*r* = 0.07, *p* < .01). The table in Appendix B presents a correlation matrix, including descriptive statistics and correlations of all variables included in the model.

### 3.2 Main analysis

**Fig. 3:**
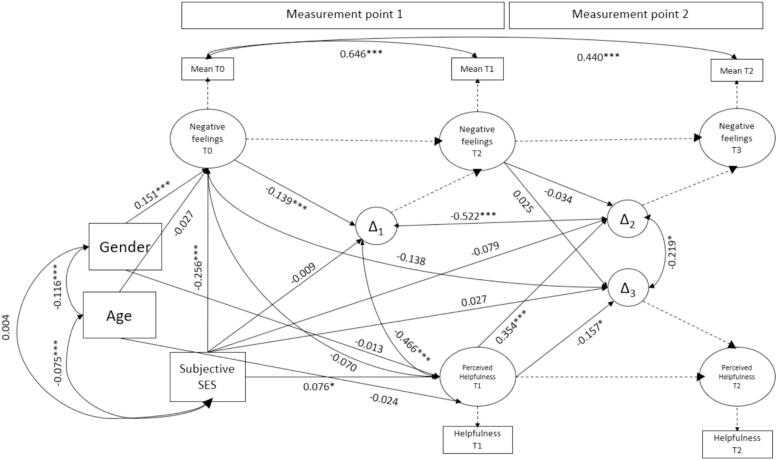
Latent change score model with all model parameters.

All results of statistical requirement tests are reported in Appendix A. The proposed latent change score model fitted the data well, χ2 (7) = 38,897, *p* < .001, CFI = 0.98, AIC = 45,277.31, SRMR = 0.03, RMSEA = 0.05, 90 %-CI = [0.03, 0.06]. All fit indices indicated a good fit (Hu and Bentler, 1999). Therefore, the model was accepted. Fig. 3 shows the full model with all estimated parameters. The table in Appendix C shows all model parameters with their respective standard error from which confidence intervals can be derived. The significant latent change score variances revealed substantial individual differences regarding the change in negative feelings and helpfulness, indicating a high level of heterogeneity in change among all participants. Gender was associated with the extent of negative feelings at T0 (*r* = 0.15, *z* = 3.49, *p* < .001). Age was negatively correlated with SES, indicating that older participants rated their SES worse, but the relationship remained small (*r* = −0.08, *z* = −3.43, *p* = .001).

Hypothesis 1 SES was negatively associated with the baseline of negative feelings (*r* = −0.26, *z* = −6.34, *p* < .001). Therefore, the lower the SES, the more negative feelings were reported before the chat.

Hypothesis 2 (***a*** + ***b***) All other regression coefficients of SES on the change scores T0-T1 and T2-T1 remained non-significant, meaning that SES was not directly associated with the change in negative feelings over time.

Hypothesis 3 (***a*** + ***b***) SES was positively correlated with the perceived helpfulness directly after the chat (*r* = 0.08, *z* = 2.90, *p* = .004). Thus, children from higher SES backgrounds rated krisenchat as more helpful than those from lower SES backgrounds. However, SES was not associated with the change regarding the helpfulness ratings.

**Explorative Analysis**. The proportional change parameter β1 was significant, indicating that the amount of change between the first and second measurement point depends on the latent baseline of negative feelings (*r* = −0.14, *z* = −14.33, *p* < .001). Thus, higher ratings of negative feelings came with less change in negative feelings. The coupling parameter γ_2_ was significant, meaning that higher ratings of perceived helpfulness were associated with more change in negative feelings from directly after the chat to four weeks later (*r* = 0.35, *z* = 8.17, *p* < .001). Thus, participants who had rated krisenchat to be more helpful previously, perceived more negative feelings again four weeks after the chat.

In addition, the perceived helpfulness directly after the chat correlated negatively with the first change score (*r* = −0.47, *z* = −15.93, *p* < .001), indicating that those, whose negative emotions diminished more between the first two measurement points, rated krisenchat as more helpful. The latent change scores of negative feelings correlated significantly negative with each other (*r* = −0.52, *z* = −5.34, *p* < .001). Thus, an improvement in negative feelings during the chat was associated with a worsening of those feelings four weeks later.

**Fig. 4:**
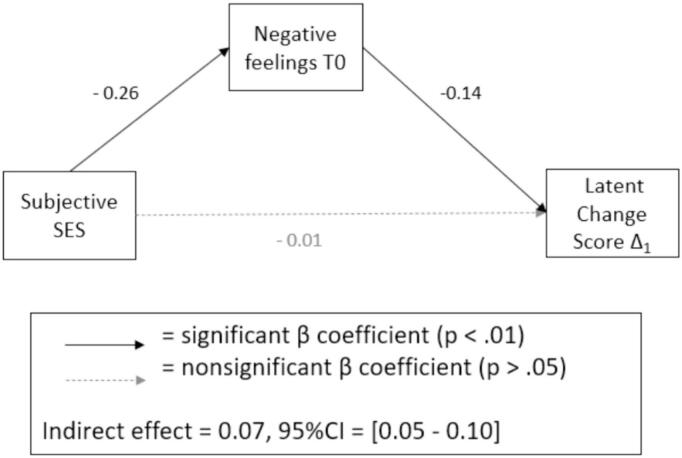
Mediation Model. Abbrev: CI = confidence interval; SES = Socioeconomic Status.

***Mediation Analysis***. Both paths, from SES to negative feelings and from negative feelings at T0 to the first latent change score Δ1 were significant. To check whether this relationship was robust, a mediation analysis with 1000 bootstrap intervals was conducted in *lavaan*. The significant parameter estimate (*β* = 0.07) and the confidence interval ranging above zero (95 % CFI = [0.05–0.10]) indicate a significant indirect effect of SES on the first change score, which is mediated by the baseline negative feelings at T0. The mediation model is shown in Fig. 4.

## 4 Discussion

The data from children and youth engaging with the German chat-based online counseling service krisenchat, were analyzed using a latent change score model. In the sample, SES was not directly associated with the change in negative feelings at any point in time. Thus, the online counseling program offers a feasible low-threshold service for children and youth from all social classes. Nevertheless, further exploratory analysis uncovered a mediating effect of SES on the evolution of negative feelings throughout the counseling session. In other words, online counseling is somewhat less effective in regulating negative emotions in children and youth from low SES backgrounds as compared to those from higher SES backgrounds. This interpretation is supported by the finding that children from families with low SES perceived krisenchat as less helpful than their counterparts with higher SES directly after the chat. However, these children, who experience more negative emotions, have a higher chance of suffering from severe mental disorders. Since krisenchat aims at short-term emotion regulation and self-reliance, it cannot treat mental disorders like online therapy interventions. This may be reflected in the lower ratings by children and youth from families with low SES.

The cycle of mental health and low SES is challenging to disrupt due to its systemic nature, which extends beyond individual control. Online counseling of the youth can neither help a family's financial hardship nor solve problematic parenting behaviors, such as inattentive or hostile parenting (Conger and Conger, 2002; Kiadarbandsari et al., 2016). Systematic approaches addressing the whole family system may be more rewarding under the circumstances of low SES. Nevertheless, providing a low-threshold help-service accessible to disadvantaged youth furthering their self-reliance and adding positive experiences with (public) health-services can add to their development beyond the relatively short-term emotion regulation investigated here.

Online counseling may reach societal and institutional barriers when trying to reach and counsel children from low SES backgrounds. Because of heightened systemic barriers like stigmatization, a lack of mental health literacy, and negative family beliefs regarding help services in low-SES families (Baldofski et al., 2023). Moreover, immigrant status, impeded oral and educational development among low-SES children (Leong et al., 2013a; Farkas and Beron, 2004a) may affect their ability to contact a chat-based online counseling service. Immigrant status is a risk factor for discrimination, acculturative stress, family conflict, and low SES (Leong et al., 2013b). Immigrant children often do not speak German at home (Scheele et al., 2010). Moreover, living in a household where both parents are from the working class affects oral vocabulary in children from early childhood on (Farkas and Beron, 2004b). In addition, children and young people from minoritized ethnic backgrounds face structural inequalities in mental health treatment settings (Ruphrect-Smith et al., 2024).

Furthermore, counselors of high education may fail to understand the circumstances and needs of children and youth from low SES families (Williams et al., 2010; Constantine, 2002). As well as counseling clients from different cultural backgrounds, counseling children and youth from different socioeconomic backgrounds acquires specialized knowledge of socially induced problems and barriers, a high amount of empathy, and an understanding of one's social position and the affiliated privileges (Constantine, 2002). Although the needs of children and youth from low SES families have been researched to some extent (Ramburuth and Härtel, 2010; Zilberstein, 2016), not all counselors may be equipped with this knowledge.

### 4.1 Limitations and strengths

Several limitations must be taken into account when looking at this study. First, the significant difference between completers and non-responders regarding age and gender may have affected the model parameter. However, these are only control variables, other important predictors, such as SES and baseline level of negative feelings did not differ significantly. Moreover, in the second measurement point, just about one third of the sample took part in the study. Additionally, self-selection, i.e., respondents recruiting themselves into the study rather than being selected, leads to biased estimates if uncorrected (Mercer et al., 2017). In the feedback questionnaire, negative feelings before the chat were assessed retrospectively, which could have skewed the data due to recall bias (Blome and Augustin, 2015), or led to inconsistent measurement errors (Hipp et al., 2020). Thus, even though this study used a causal model of analysis, all results should be interpreted with caution. In addition, disclosing one's SES was not mandatory, which is critical when considering that low SES individuals are more likely not to disclose their SES out of shame (MacDonald and Morley, 2001; Farber, 2003). Moreover, the item asked a complex question, where participants had to compare themselves to the abstract Germany society, which is rather difficult to answer. The item itself was not validated and used a coarse four-point scale without midpoint. SES is a measure that needs some level of cognitive abilities and an understanding of the abstract concept of “other families in Germany, which I do not know.” Nevertheless, a study by Amir and colleagues from 2019 demonstrated that children from different cultures could reliably judge their SES based on the McArthur Scale of Subjective Socioeconomic Status from age four (Amir et al., 2019).

The STOP-D Items were rated on a slider, which was randomly placed either at 0 or 9. Thus, if a participant answered an item with either a 0 or 9, it is not clear whether they chose 0 or 9 as an item answer, or simply did not respond to the item. The study only used negative emotions as outcome measures next to the perceived helpfulness. Positive emotions or during the counseling process acquired skills were not assessed. A lot of users successfully overcome their crisis with their own strengths or minimal guidance from the krisenchat counselors.

Regardless of these limitations, the study also has considerable strengths. It was the first study to examine the relationship between SES and counseling success in a context of online counseling for children and youth. It was conducted based on a sample of >2000 participants recruited from real-life online counseling and used advanced statistical methods on longitudinal data to analyze proposed relationships. It provided new insights into the disadvantages that children and youth from low SES families experience and has shown that there still is a long way to go until equal access to crisis support is reached for all children and youth in Germany.

### 4.2 Further research

To reach children from low SES families as adequately as their higher SES counterparts, we need to know the mechanisms behind the complex relationship between SES and online counseling success. Further research should address the following questions, possibly through qualitative studies or longitudinal designs: What do children from low SES families need from counselors? How can barriers be reduced? An explicit look at referral success as a marker for counseling success rather than the reduction of negative feelings might be fruitful. Baldowski et al. already showed that successful help-seeking behavior was associated with self-efficacy (Baldofski et al., 2023). Thus, it may be feasible to directly target and increase self-efficacy during the counseling session. This may be especially important for children and youth from low SES backgrounds.

## 5 Conclusions

This study has shown that children and youth from low SES backgrounds suffer from more negative feelings, have a higher need for counseling services and yet do not profit to the same extent from existing services as more privileged youth. Online counseling may not be able to change the disadvantageous and hostile environments children and youth from low SES families find themselves in every day. Still, online counseling is not useless. It is accessible for children and youth from all social classes, offers emotional support, and fosters inherent resilience. SES has a profound impact on the developmental trajectory and health outcomes of children and youth. The earlier children receive support despite their disadvantageous socioeconomic circumstances the better later health problems can be prevented.

## CRediT authorship contribution statement

The following statements should be used Conceptualization, F.R.; methodology, F.R., J.T. and M.Z.; formal analysis, F.R.; resources, J.T. and M.Z.; data curation, J.T.; writing—original draft preparation, F.R.; writing—review and editing, J.T.; F.R., A.B., C.RK., G.M., L.K., R.W. and M.Z.; visualization, F.R; project administration, krisenchat.; funding acquisition, krisenchat.

## Funding

This work was supported by The German 10.13039/501100003107Federal Ministry of Health (ZMI1-2521FEP001).

## Institutional review board statement

The analysis in this study was a secondary data analysis which received ethical by the Institutional Review Board (or Ethics Committee) of Medical University of Leipzig (372/21-ek with reference to the addendum dated 6.7.2022) (Ethikkommission der Medizinischen Universität Leipzig, (ZMI1-2521FEP001)).

## Declaration of competing interest

AB, LK, and MZ confirm no conflicts of interest. JT, and RW are paid employees of krisenchat gGmbH. FR is a volunteer at krisenchat gGmbH. GM received funding from the Stanley Thomas Johnson Stiftung & Gottfried und Julia Bangerter-Rhyner-Stiftung under projects no. PC 28/17 and PC 05/18, from Gesundheitsförderung Schweiz under project no. 18.191/K50001, from the Swiss Heart Foundation under project no. FF21101, from the Research Foundation of the International Psychoanalytic University (IPU) Berlin under projects no. 5087 and 5217, from the Swiss National Science Foundation (SNSF) under project no. 100014_135328, from the Hasler Foundation under project No. 23004, in the context of a Horizon Europe project from the Swiss State Secretariat for Education, Research and lnnovation (SERI) under contract number 22.00094, and from Wings Health in the context of a proof-of-concept study. Further, he is a co-founder, member of the board, and shareholder of Therayou AG, active in digital and blended mental healthcare. He received royalties from publishing companies as author, including a book published by Springer, and an honorarium from Lundbeck for speaking at a symposium. He is compensated for providing psychotherapy to patients, acting as a supervisor, serving as a self-experience facilitator (‘*Selbsterfahrungsleiter*’), and for postgraduate training of psychotherapists, psychosomatic specialists, and supervisors. CRK received lecture honoraria from Recordati and Servier outside and independent of the submitted work.

## Figures and Tables

**Table A1:** Results of Shapiro-tests.

Variable	W	*p*
Mean T0	0.971	<0.001
Mean T1	0.978	<0.001
Mean T2	0.960	<0.001
Helpfulness T1	0.764	<0.001
Helpfulness T2	0.789	<0.001

**Table A2:** Results of Levene-tests.

Variable	F (1,641)	*p*
Age	10.097	<0.001
Gender	11.201	<0.001
SES	9.547	0.002
Mean T0	0.991	0.319

**Table B1:** Means, standard deviations, and correlations with confidence intervals.

Variable	*M*	*SD*	1	2	3	4	5	6	7
**Negative feelings**									
1. T0	6.40	1.56							
2. T1	4.46	2.25	0.46**						
			[0.43, 0.49]						
3. T2	5.85	1.96	0.40**	0.32**					
			[0.34, 0.45]	[0.26, 0.38]					

**Perceived helpfulness**									
4. T1	2.34	0.72	−0.04	−0.33**	−0.11**				
			[−0.08, 0.01]	[−0.37, −0.29]	[−0.18, −0.04]				
5. T2	2.25	0.72	−0.06	−0.28**	−0.20**	0.60**			
			[−0.13, 0.01]	[−0.35, −0.21]	[−0.27, −0.13]	[0.55, 0.65]			

**Demographic data**									
6. subjective SES	2.88	0.86	−0.13**	−0.07**	−0.10**	0.07**	0.06		
			[−0.17, −0.09]	[−0.12, −0.03]	[−0.17, −0.04]	[0.03, 0.11]	[−0.01, 0.13]		
7. Gender			0.07**	0.00	0.19**	0.01	−0.06	0.00	
			[0.03, 0.12]	[−0.04, 0.05]	[0.13, 0.26]	[−0.03, 0.06]	[−0.14, 0.01]	[−0.04, 0.05]	
8. Age	16.93	3.37	−0.01	0.00	−0.08*	−0.03	0.08*	−0.08**	−0.12**
			[−0.05, 0.03]	[−0.04, 0.04]	[−0.14, −0.01]	[−0.08, 0.01]	[0.01, 0.15]	[−0.12, −0.03]	[−0.16, −0.07]

*Note*. M and SD are used to represent mean and standard deviation, respectively. Values in square brackets indicate the 95 % confidence interval for each correlation. The confidence interval is a plausible range of population correlations that could have caused the sample correlation. * indicates *p* < .05. ** indicates *p* < .01. Abbrev.: SD, standard deviation; SES, socioeconomic status.

**Table C1:** All standardized model parameters with standard error.

Parameter and fit indices	Model	
	Parameter	SE
Negative feelings T1 → Delta feelings T1-T0 (β_1_)	−0.139***	0.021
Negative feelings T1 → Delta feelings T2-T1 (β_2_)	−0.034	0.046
Perceived helpfulness T1 → Delta helpfulness T2-T1 (β_3_)	−0.157	0.033
Negative feelings T1 → Delta helpfulness T2-T1 (γ_1_)	0.025	0.011
Perceived helpfulness T2 → Delta feelings T2-T1 (γ_2_)	0.354***	0.104
**Regressions**		
**Age**		
→ Negative feelings T0	−0.027	0.011
→ Perceived helpfulness T1	−0.024	0.004
**Gender**		
→ Negative feelings T0	0.151***	0.077
→ Perceived helpfulness T1	0.013	0.028
**SES**		
→ Negative feelings T0	−0.256***	0.039
→ Perceived helpfulness T1	0.076**	0.019
→ Delta feelings T1-T0	−0.009	0.045
→ Delta feelings T2-T1	−0.079	0.084
→ Delta helpfulness T2-T1	0.027	0.026
**Latent change score variances**		
Delta feelings T1-T0	0.981***	0.242
Delta feelings T2-T1	0.830***	0.593
Delta helpfulness T2-T1	0.973***	0.074
**Error variances**		
Observed feelings T0	8.216***	0.263
Observed helpfulness T1	3.491***	0.114
**Covariances**		
**Mean T0**		
→ Mean T1	0.646***	0.087
→ Mean T2	0.440***	0.110
**Control variables**		
Gender → age	−0.116***	0.032
Gender → SES	0.004	0.009
Age → SES	−0.075**	0.063
**Negative feelings T1**		
→ Perceived helpfulness T2	0.070	0.026
→ Delta helpfulness T2-T1	−0.128	0.031
**Delta feelings T1**-**T0**		
→ Perceived helpfulness T2	−0.466***	0.032
→ Delta feelings T2-T1	−0.522***	0.233
**Delta feelings T2**-**T1**		
→ Delta helpfulness T2-T1	−0.219**	0.042
**Fit indices**		
Parameters	40	
Degrees of freedom	6	
Chi-square value	40748***	
RMSEA [90-CFI]	0.047 [0.034–0.062]	
CFI	0.976	
SRMR	0.034	
AIC	45,277.307	

*Note*: CFI = Comparative Fit Index; AIC = Akaike information criterion; β = proportional change coefficient. Parameters not reported here were fixed to zero (for variances) or 1 (for loadings and means) * *p* < .05. ** *p* < .01. *** *p* < .001. Abbrev.: SES = Socioeconomic Status, RMSEA = Root Mean Square Error of Approximation, SRMR = Root Mean Square Residual.

## Data Availability

Restrictions apply to the availability of these data. Data were obtained from krisenchat gGmbH.

## Appendix A

Shapiro-Tests revealed that none of the used variables followed a normal distribution. Even though a violation of the assumption of normally distributed data is not optimal, the model should be robust enough to proceed with the analysis when using the robust maximum likelihood estimator. Levene-Tests established homoscedasticity of variances between non-responders to T2 and completers, so drop-out selectivity could be tested. Results of both Shapiro and Levene-Tests are shown in Table A1, Table A2. Thus, ANOVAs for SES, baseline of negative feelings and age, as well as a Chi-Square-Test for gender were conducted and revealed no significant differences between the composition of completers and non-responders regarding SES, *F*(1, 2137) = 2.46, *p* = .117, ƞ2 < 0.001. The analyses also revealed no significant differences regarding the baseline mean of negative feelings, *F*(1, 2137) = 0.93, *p* = .335, ƞ2 < 0.001. However, completers differed significantly from non-responders regarding age, with non-responders being on average one year older than completers, *F*(1, 2137) = 25.92, *p* < .001, ƞ2 < 0.012 and gender, *X*^*2*^(2, 2137) = 11.367, *p* = .003), which is most likely due to the high gender imbalance in the sample.
